# Sonographic Assessment of Axillary Lymph Nodes Post COVID-19 Vaccine

**DOI:** 10.7759/cureus.48630

**Published:** 2023-11-10

**Authors:** Afnan Almuhanna, Zainab S AlTurifi, Shaima A Bin Saad, Fatimah T Albaqshi, Nora A Almomen, Walaa Y Almuhanna, Buthaynah M Almuhaini

**Affiliations:** 1 Radiology, King Fahad University Hospital, Khobar, SAU; 2 Radiology, Imam Abdulrahman Bin Faisal University, Khobar, SAU

**Keywords:** covid-19 vaccine, lymph nodes, ultrasound, lymphadenopathy, covid-19

## Abstract

Background

The development of axillary lymphadenopathy post COVID-19 vaccine became an area of concern. This needs to be addressed and investigated to provide updated data that could contribute to its management and imaging guidelines.

Objectives

This study aims to detect possible changes in lymph nodes (LNs) after COVID-19 vaccination, decrease the rate of over-investigation and medical costs, and identify possible predisposing factors that could trigger the development of lymphadenopathy.

Methods

This was a retrospective cohort study conducted in King Fahad University Hospital, a secondary hospital in Al Khobar, Kingdom of Saudi Arabia (KSA), on a total of 1,257 vaccinated patients who underwent axillary ultrasonography (US) from December 2020 till the second of October 2022. All required data were collected using an Excel sheet and analyzed using Statistical Product and Service Solutions (SPSS, version 21) (IBM SPSS Statistics for Windows, Armonk, NY).

Results

Out of the 1183 cases, 460 (38.9%) cases had abnormal LNs post COVID-19 vaccine based on US, while LNs were normal in 723 (61.1%) cases. The prevalence of axillary lymphadenopathy was significantly more common in females than males (39.1% of cases in female patients versus 22.2% of cases in male patients (P = 0.049)). In addition, lymphadenopathy was more prevalent among patients who received the COVID-19 vaccine less than four weeks ago, compared to others who received the vaccine four to eight weeks ago, 8-12 weeks ago, and more than 12 weeks ago (100% vs 73.3% vs 34.2% vs 72% (P = 0.001)).

Conclusion

In conclusion, a significant number of patients were found to have lymphadenopathy after receiving the COVID-19 vaccine. The only predisposing factors identified to be associated with the development of lymphadenopathy were gender (females more than males) and duration since receiving the vaccine (four weeks).

## Introduction

In December 2019, in Wuhan, Hubei province, China, a series of pneumonia cases of unknown cause were reported, which the Chinese Center for Disease Control and Prevention (China CDC) described as novel coronavirus [1]. The WHO named the disease coronavirus disease 2019 (COVID-19) and the virus as severe acute respiratory syndrome coronavirus 2 (SARS-CoV-2) in February 2020. Since then, COVID-19 spread all over the world, and it was declared by WHO as a pandemic in March 2020 [2,3]. The first case of COVID-19 reported in Saudi Arabia was on the second of March 2020 [4].

The Kingdom of Saudi Arabia (KSA) approved BNT162b2 mRNA (Pfizer-BioNtech) vaccine in December 2020 and started administering the vaccine in January 2021. Other vaccine types approved and used in Saudi Arabia included ChAdOx1 nCoV-19 (AZD1222) of the Oxford-AstraZeneca, Moderna, and Janssen [5,6].

With COVID-19 vaccination becoming prevalent worldwide, vaccine-related side effects started to show, one of which is lymphadenopathy. This became a major issue for both clinicians and patients. Lymphadenopathy was observed in the ipsilateral axilla to the vaccine site, and this increased the concern about underdiagnosis or overdiagnosis along with patient anxiety [7].

Identifying the characteristics of vaccine-related lymphadenopathy would be very beneficial in decreasing the diagnostic burden of it being misdiagnosed as cancer, which would lead to providing the patient with unsuitable treatment for their condition; determining the appropriate time or duration for a patient to undergo imaging before and after receiving the vaccine; and finally recognizing when to send the patient for further diagnostic studies, such as biopsy and FNAC [8-12].

Many limitations have been found in different studies, including the lack of follow-ups, biopsies with pathological correlation, type of vaccine, rate of lymphadenopathy, size and number of affected lymph nodes (LNs), and small sample size [13-16].

The primary aim of this retrospective study is to investigate if there is a certain pattern of axillary lymphadenopathy on ultrasonography (US) following COVID-19 vaccination, confirmed by biopsy. In addition, the study aims to determine if there are any biological factors that could increase the possibility of developing lymphadenopathy in both men and women aged 20-70 post vaccination. The findings from this study will help in the prevention of over-screening and the investigation of patients presenting with lymphadenopathy following the COVID-19 vaccine.

## Materials and methods

Study setting and design

This is a retrospective cohort study that was conducted at King Fahad University Hospital, a secondary hospital in Al Khobar, KSA.

Subjects

The study included a total of 1,257 vaccinated patients who underwent US from December 2020 to the second of October 2022. Patients’ demographic data, biological biography, LN changes, COVID-19 vaccine information, biopsy results, and follow-ups were all collected in an Excel sheet for analysis. Table 1 provides the inclusion and exclusion criteria of the subjects.

**Table 1:** Inclusion and exclusion criteria

Inclusion criteria	Exclusion criteria
Saudis and non-Saudis	Patient diagnosed with breast inflammatory disease
Patients of 20 years old and up to 70 years old	Patients under 20 years old
Patient that underwent US	Patients over 70 years old

Procedure

Data were collected from imaging findings in the breast and axillary regions using the ultrasound RS85 Samsung, a high-frequency, linear probe, 11-megahertz, grey on-scale machine (Samsung Electronics Co., Ltd., Suwon, South Korea).

The study approval was obtained from Imam Abdulrahman bin Faisal University Institutional Review Board on 30/10/2022 (IRB-UGS-2022-01-411). Consent was not required since the data were collected from the hospital records and not directly from the patients.

Data analysis

The data were collected, reviewed, and then fed to Statistical Product and Service Solutions (SPSS) (IBM SPSS Statistics for Windows, Armonk, NY). All statistical methods used were two-tailed, with an alpha level of 0.05, considering significance if the P-value is less than or equal to 0.05 (P ≤ 0.05).

Descriptive analysis was done by prescribing frequency distribution and percentage for study variables, including participants' personal data, personal and family history data, and co-morbidities. Additionally, the sonographic findings in the LNs were tabulated, and the frequency and pattern of abnormal LNs were graphed. The data were represented as an absolute number and percentages. Furthermore, cross tabulation was performed to show factors associated with abnormal LNs among the study cases using the Pearson chi-square test for significance and exact probability test if there were small frequency distributions. The results are reported to be statistically significant whenever P < 0.05.

## Results

A total of 1,183 cases that met the inclusion criteria were included in this study. Patients’ ages ranged from 20 to 70 years, with a mean age of 46.5 ± 12.9 years old; 1,165 (98.5%) patients were female, and 1,018 (86.1%) were Saudi (Table 2). As for personal history, 101 (8.5%) of the study participants had breast malignancy, 22 (1.9%) had a history of inflammatory diseases such as scleroderma and systemic lupus erythematosus, and the vast majority (1,161, 88.5%) had no past history. A total of 119 (10.1%) patients had a family history of breast cancer, while 336 (28.4%) had other co-morbidities such as diabetes mellitus and hypertension. With regard to COVID-19 vaccine duration, it was 8-12 weeks ago among 1,040 (88%) cases, more than 12 weeks ago among 125 (10.6%) cases, and four weeks ago or less among two (0.2%) cases.

**Table 2:** Bio-demographic data of study patients who received the COVID-19 vaccine, King Fahad University Hospital, Al Khobar, KSA

Personal data	No	%
Age in years		
20-30	223	18.9%
30-40	320	27.0%
40-50	327	27.6%
50-60	222	18.8%
60-70	91	7.7%
Gender		
Male	18	1.5%
Female	1165	98.5%
Nationality		
Saudi	1018	86.1%
Non-Saudi	165	13.9%
Personal history		
None	1047	88.5%
Breast malignancy	101	8.5%
Breast inflammatory	22	1.9%
Breast abscess	9	0.8%
Lymphatic	4	0.3%
Family history		
Yes	119	10.1%
No	1064	89.9%
Co-morbidities		
Yes	336	28.4%
No	847	71.6%
COVID-19 vaccine		
Less than 4 W	2	0.2%
4-8 W	15	1.3%
8-12 W	1040	88.0%
More than 12 W	125	10.6%

For the LN assessment among patients who received the COVID-19 vaccine at King Fahad University Hospital, Al Khobar, KSA, a total of 39 (3.3%) of the study patients had LNs more than 1.5 cm (Table 3). As for morphology, it was oval among 636 (53.8%) patients, round among 296 (25%) patients, but irregular among 239 (20.2%) and speculated among 12 (1%) cases. Hilum was absent among 179 (15.1%) cases. Cortex D3 was more than 0.3 cm among 310 (27.1%) of the study cases. Hypervascularity was detected among 240 (20.3%) cases. Ipsilateral lymphadenopathy was found in 700 (59.2%) cases. In addition, 143 (12.1%) patients underwent a biopsy, and there were 55 (38.7%) positive cases.

**Table 3:** Lymph node assessment with biopsy among patients who received the COVID-19 vaccine, King Fahad University Hospital, Al Khobar, KSA

Lymph node	No	%
Lymph node size		
Below 1.5 cm	1144	96.7%
1.5-2 cm	33	2.8%
Above 2 cm	6	0.5%
Lymph node morphology		
Oval	636	53.8%
Round	296	25.0%
Irregular	239	20.2%
Speculated	12	1.0%
Hilum		
Yes	1004	84.9%
No	179	15.1%
Cortex D3		
Below 0.3 cm	862	72.9%
0.3-0.5 cm	261	22.1%
0.5-1 cm	49	4.1%
Above 1 cm	11	0.9%
Vascularity		
Yes	240	20.3%
No	943	79.7%
Ipsilaterally		
Yes	700	59.2%
No	483	40.8%
Biopsy		
Yes	143	12.1%
No	1040	87.9%
Pathology		
Positive	55	38.7%
Negative	87	61.3%

**Figure 1:**
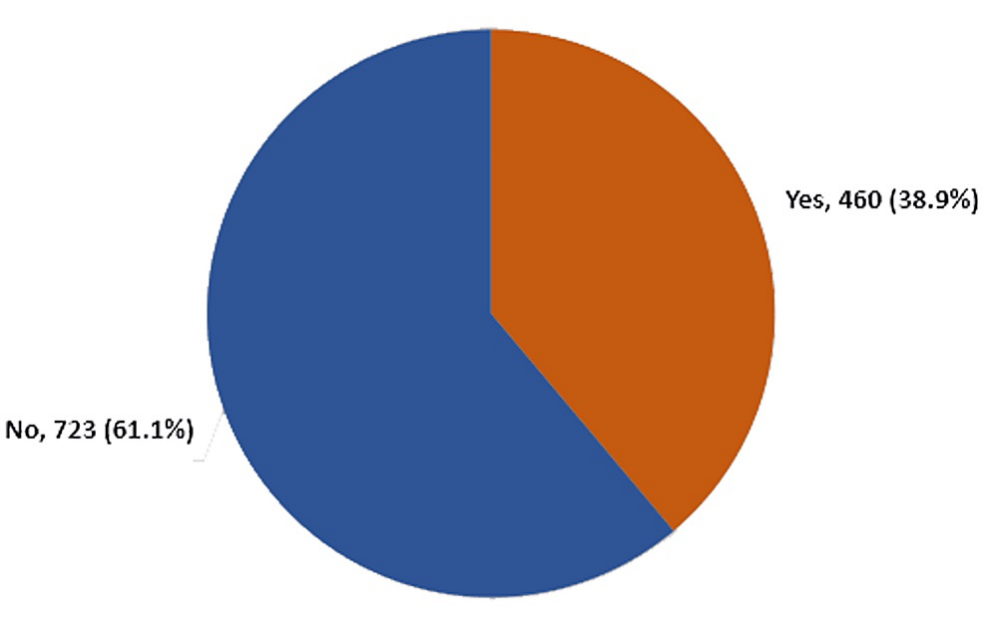
Abnormal lymph nodes among patients who received COVID-19 vaccine at King Fahad University Hospital, Al Khobar, KSA

For the abnormal LNs among patients who received the COVID-19 vaccine at King Fahad University Hospital (Figure 1), Al Khobar, KSA, a total of 460 (38.9%) cases had abnormal LNs based on US, while LNs were normal among 723 (61.1%) patients.

The parameters used in this study to categorize LNs as either normal or abnormal include morphology, size, cortical thickness, presence or absence of central hilum, and vascularity. Normal LNs are characterized by an oval appearance, with cortical thickness ≤ 3 mm and a preserved fatty hilum. Emphasis is placed on uniformity and cortical thickness as the two most important characteristics differentiating normal and abnormal LNs [17].

Table 4 presents the sonographic pattern of abnormal LNs among patients who received the COVID-19 vaccine at King Fahad University Hospital, Al Khobar, KSA (n = 460). Up to 39 (8.5%) of cases with abnormal LNs had lymph node size more than 1.5 cm. Additionally, LNs were irregular among 127 (27.6%) patients, as well as with no hilum among 179 (38.9%) and with hypervascularity among 240 (52.2% cases). Likewise, cortex D3 was more than 0.3 cm among 321 (69.8%) of the cases with abnormal LNs.

**Table 4:** Sonographic pattern of abnormal lymph nodes among patients who received the COVID-19 vaccine, King Fahad University Hospital, Al Khobar, KSA (n=460)

Pattern	No	%
Lymph node size		
Below 1.5 cm	421	91.5%
1.5-2 cm	33	7.2%
Above 2 cm	6	1.3%
Lymph node morphology		
Oval	201	43.7%
Round	122	26.5%
Irregular	127	27.6%
Speculated	10	2.2%
Hilum		
Yes	281	61.1%
No	179	38.9%
Cortex D3		
Below 0.3 cm	139	30.2%
0.3-0.5 cm	261	56.7%
0.5-1 cm	49	10.7%
Above 1 cm	11	2.4%
Vascularity		
Yes	240	52.2%
No	220	47.8%

Factors associated with abnormal LNs among patients who received the COVID-19 vaccine at King Fahad University Hospital, Al Khobar, KSA (Table 5). Only patients’ gender and duration since being vaccinated showed a significant association with having abnormal LNs. Abnormal LNs were detected among 456 (39.1%) of females versus 4 (22.2%) of male cases (P = 0.049). Additionally, all cases who received the vaccine recently (less than four weeks) showed abnormal LNs compared to 11 (73.3%) of others who received the vaccine four to eight weeks ago and 356 (34.2%) of the others who received the vaccine 8-12 weeks ago. Up to 90 (72%) of cases who were vaccinated more than 12 weeks ago also showed abnormal LNs (P = 0.001).

**Table 5:** Factors associated with abnormal lymph nodes among patients who received the COVID-19 vaccine, King Fahad University Hospital, Al Khobar, KSA

Factors	Abnormal LNs				P-value
	Yes		No		
	No	%	No	%	
Age in years					0.395^$^
20-30	77	34.5%	146	65.5%	
30-40	126	39.4%	194	60.6%	
40-50	137	41.9%	190	58.1%	
50-60	89	40.1%	133	59.9%	
60-70	31	34.1%	60	65.9%	
Gender					0.049*
Male	4	22.2%	14	77.8%	
Female	456	39.1%	709	60.9%	
Nationality					0.177
Saudi	388	38.1%	630	61.9%	
Non-Saudi	72	43.6%	93	56.4%	
Personal history					0.166^$^
B Abscess	6	66.7%	3	33.3%	
B Inflammatory	11	50.0%	11	50.0%	
B Malignancy	40	39.6%	61	60.4%	
Lymphatic	3	75.0%	1	25.0%	
No	400	38.2%	647	61.8%	
Family history					0.460
Yes	50	42.0%	69	58.0%	
No	410	38.5%	654	61.5%	
Co-morbidities					0.253
Yes	122	36.3%	214	63.7%	
No	338	39.9%	509	60.1%	
Duration since vaccination (weeks)					0.001*^$^
Less than 4 W	2	100.0%	0	0.0%	
4-8 W	11	73.3%	4	26.7%	
8-12 W	356	34.2%	684	65.8%	
More than 12 W	90	72.0%	35	28.0%	

## Discussion

In 2019, an outbreak of pneumonia of unknown cause disseminated in Wuhan, China, causing a disease later named COVID-19. This disease spread all over the world and was announced as a pandemic by the WHO. Saudi Arabia was one of the countries affected by the virus, and the first case was reported on March 2, 2020. Since then, the Saudi government has taken the necessary measures and precautions regarding the early detection and management of COVID-19 cases, health promotion, and consideration for healthcare providers and communities, as well as strict measures for quarantine [1-4,18]. In April 2020, the Ministry of Health initiated virtual clinics to limit the need for hospital visits, in addition to home delivery of medication. Furthermore, in June 2020, the efforts of the Kingdom kept increasing, as evidenced by the establishment of Tetamman clinics all over the Kingdom to provide services and medical consults without the need to visit the emergency department in hospitals. One critical decision was limiting Hajj in 2020 due to the pandemic, which occurs once a year on specific days, on which Muslims around the world come to perform the holy rites and amulets. In December 2020, registration for receiving COVID-19 vaccine was available. As of April 2022, registration was open in the Sehhaty app for the second dose, prioritizing individuals who were 50 years and older. As of July 2022, registration for the third booster dose was available in the app, prioritizing the older age group as well [19].

The KSA adopted vaccination and provided it for all citizens and immigrants for free. The Pfizer-BioNTech vaccine was approved in December 2020 and administered in January 2021 in two doses, with a maximum interval of 42 days. This vaccine was dedicated to 12-year-olds and above. Other vaccines included Oxford-AstraZeneca, which covered 18-year-olds and above in two doses, with an interval of 12 weeks. Moderna was another variant of the COVID-19 vaccine for 12-year-olds and above, given in two doses, similar to other vaccines, with an interval of 28 days between doses [5,6,20].

Following vaccine administration, axillary lymphadenopathy was a common finding that was noticed by physicians among patients who received COVID-19 vaccination. Therefore, many recommendations have been proposed by the European Society of Breast Imaging (EUSOBI) to standardize the management of the adverse effects and prevent unnecessary investigations and procedures in such patients (Table 6) [21].

**Table 6:** European Society of Breast Imaging (EUSOBI) recommendations Source: Ref [21]

European Society of Breast Imaging (EUSOBI) recommendations:	
Vaccines should be received in the opposite arm or in the thigh in patients with a positive history of breast cancer.	1
Vaccination data should be collected for all patients who visit breast imaging clinics to stage breast cancer and follow-up imaging examinations.	2
Breast imaging should be performed before vaccination or 12 weeks after vaccination as a minimum.	3
Standard imaging protocols should be done for patients newly diagnosed with breast cancer.	4
To exclude malignancy, appropriate imaging examination of the opposite axilla and both breasts should be done before vaccination or 12 weeks after, as a minimum, in case of symptomatic or imaging-confirmed axillary lymphadenopathy.	5
Standard workup should be done if axillary lymphadenopathy is found opposite the vaccination side.	6
Ipsilateral axillary lymphadenopathy within 12 weeks post-vaccination could be considered benign or probably benign in patients with a negative history of breast cancer, taking into consideration the clinical context.	7
Standard workup should be done for patients with a negative past history of breast cancer but with axillary lymphadenopathy, in combination with suspicious breast findings. This should include a biopsy if appropriate.	8
Regarding the interpretation and management of lymphadenopathy, the timeframe from receiving the vaccine and risk of nodal metastasis should be considered in patients with a history of breast cancer.	9
A multidisciplinary team is important in managing uncertain or complex cases.	10

In addition, Table 7 presents the recommendation of the Society of Breast Imaging regarding axillary lymphadenopathy post COVID-19 vaccination [22].

**Table 7:** The Society of Breast Imaging recommendations Source: Ref [22]

The Society of Breast Imaging recommendations:	
Data regarding the vaccine, including timing and side of vaccination, should be collected.	1
Patients with axillary lymphadenopathy on screening exams should be labeled as a BI-RADS O to give room for additional assessment	2
Short-term follow-up for 4-12 weeks after the second dose should be provided after proper diagnostics evaluation for axillary lymphadenopathy.	3
Lymph node sampling should be performed to exclude malignancy, in case of persistent axillary lymphadenopathy	4
Finally, if possible, consider screening exams prior to the first dose of a COVID-19 vaccine or 4-6 weeks following the second dose.	5

Representative cases

**Figure 2:**
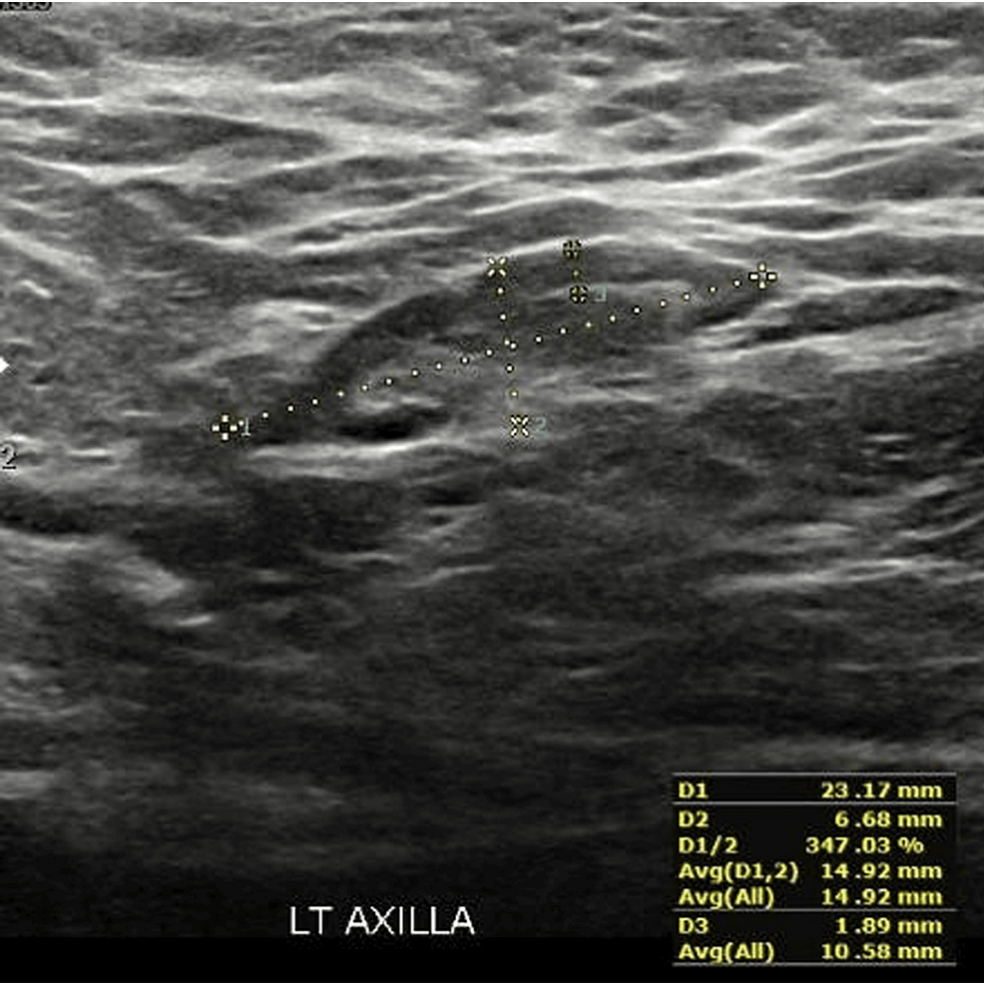
Ultrasound image showing a normal axillary lymph node This figure was obtained from the KFUH system (author's own).

Normal axillary LN: A 23-year-old female with breast fibrocystic changes underwent US of axillary LNs eight weeks post vaccination. Ultrasound showed a unilateral oval left axillary LN with preserved hilum and no vasculature, 6.68 mm in size, with a cortical thickness of 1.89 mm (Figure 2). This case was obtained from the KFUH system and is included in the study.

**Figure 3:**
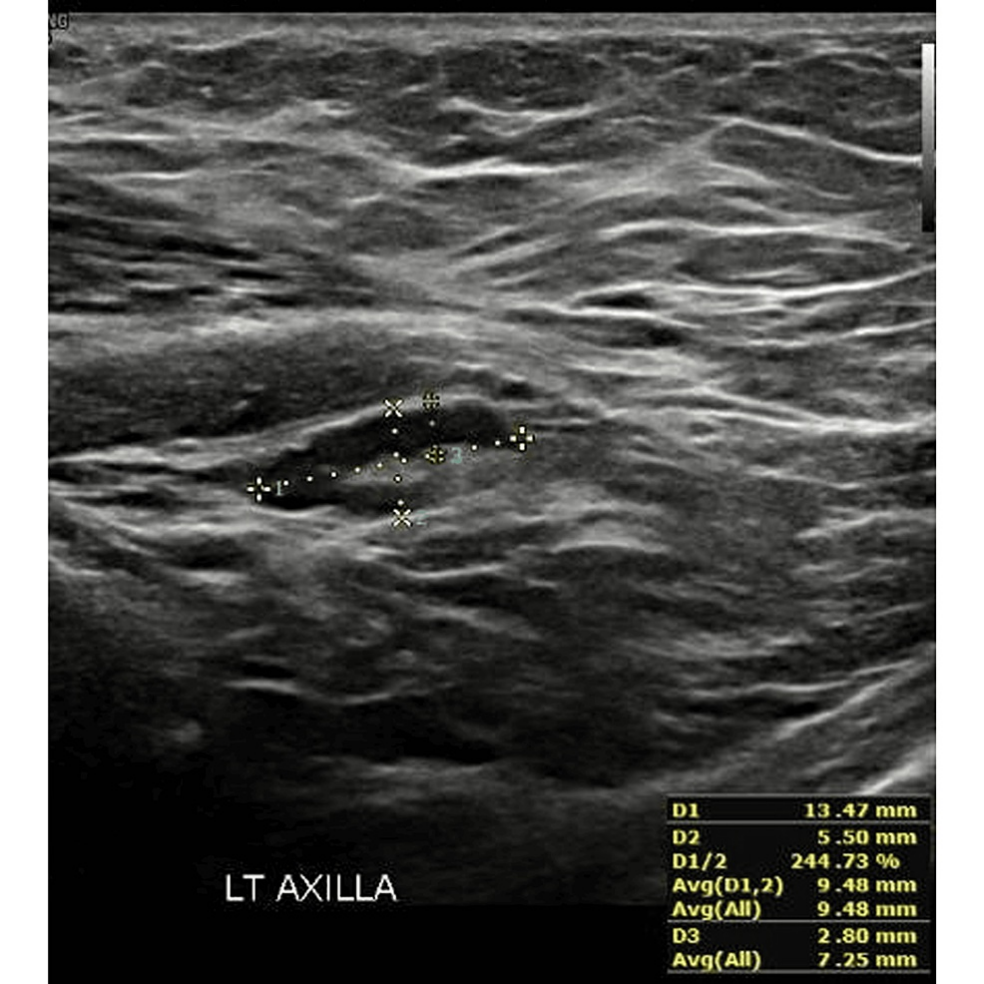
Ultrasound image showing a reactive axillary lymph node - Case A This figure was obtained from the KFUH system (author's own).

**Figure 4:**
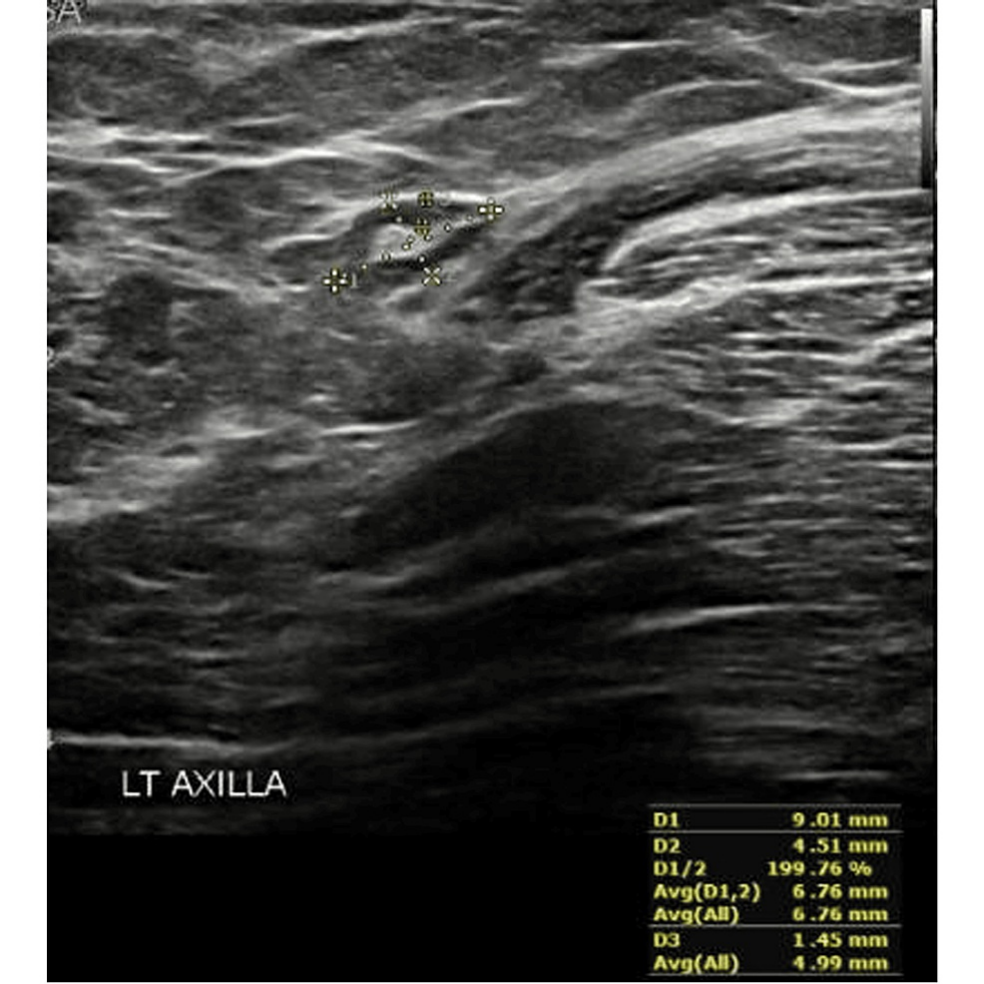
Ultrasound image showing a reactive axillary lymph node - Case B This figure was obtained from the KFUH system (author's own).

Abnormal (reactive) axillary LN: A 35-year-old female with negative breast pathology underwent US of axillary LNs eight weeks post vaccination (Figures 3, 4).

Case A. US showed a prominent left axillary LN with minimal diffuse cortical thickening that otherwise maintained central echogenic hilum measuring 13.4 x 5.5 x 2.8 mm in length, width, and cortical thickness, respectively. Case B. Twelve weeks post vaccination, axillary lymphadenopathy was resolved. This case was obtained from the KFUH system and is included in the study.

**Figure 5:**
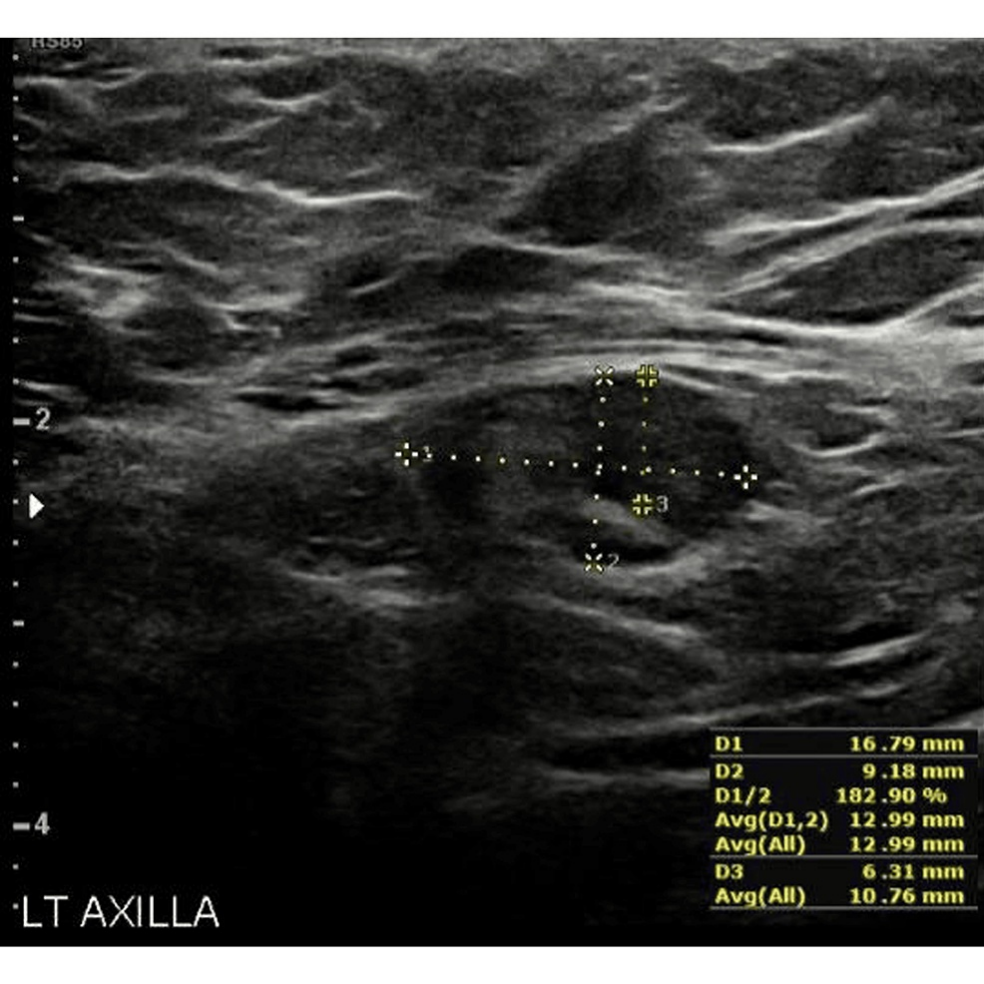
Ultrasound image of a malignant lymph node This figure was obtained from the KFUH system (author's own).

A 46-year-old woman with a known case of left breast invasive ductal carcinoma confirmed by biopsy had prominent left axillary LNs with diffuse heterogeneous cortical thickening, the largest measuring 16.8 x 9.2 mm, with cortical thickness up to 6.3 mm (Figure 5). This case was obtained from the KFUH system and is included in the study.

In this retrospective study, a total of 1,183 cases were evaluated, in which females accounted for 98.5% (1,165) of the cases, while males were only 1.5% (18 cases), with a mean age of 46.5 ± 12.9 years. The study found that, out of the 1,183 cases, 460 (38.9%) had abnormal LNs post COVID-19 vaccine based on US, while LNs were normal among 723 (61.1%). The prevalence of axillary lymphadenopathy was significantly more common in females (456 or 39.1%) than in males (four or 22.2%) (P = 0.049). In addition, lymphadenopathy was more prevalent in patients who received the COVID-19 vaccine less than four weeks ago (2 or 100%), compared to others who received the vaccine four to eight weeks ago (11 or 73.3%), 8-12 weeks ago (356 or 34.2%), and more than 12 weeks ago (90 or 72%) (P = 0.001).

Regarding the lymphadenopathy sonographic pattern post COVID-19 vaccine, 39 (8.5%) of the cases had an LN size of more than 1.5 cm compared to less than 1.5 cm (421 (or 1.5%). Furthermore, LNs were irregular in morphology in 127 cases (27.6%). In contrast, oval, round, and speculated-shaped LNs accounted for 333 (72.4%) of cases. The hilum was found to be absent in 179 (38.9%) of cases; however, it was present in 281 (61.1%). Hypervascularity was detected among 240 (52.2%) of cases, compared to 220 (47.8%) without hypervascularity. Moreover, the cortex diameter was greater than 0.3 cm among 321 (69.8%) of the cases with abnormal LNs, compared to cortex D3 less than 0.3 cm 139 (30.2%).

A total of five studies were compatible with the current study when comparing the duration aspect. For instance, a case series published in 2021 presented six cases of patients with a history of breast cancer, who presented with post COVID-19 vaccine lymphadenopathy. The study concluded that there is an association between the duration of being vaccinated and the presence of lymphadenopathy within the span of 28 days [23]. Furthermore, a study was conducted on 1,196 patients to look for lymphadenopathy after receiving COVID-19 vaccine using PET/CT. Regarding the duration, a higher prevalence was found within 20 days from the vaccine <20 days: 21/60, 45/129 (35% ) vs. 220 days: 54/248 (22%) [13]. In another study that was conducted to evaluate 433 participants for COVID-19 vaccine-related axillary lymphadenopathy, patients were evaluated in terms of days after vaccination, and a higher prevalence of lymphadenopathy was detected the in first few days post vaccination [10]. In addition, in a literature review of 68 cases, including both genders, 60 (88.2%) females and eight (11.8%) males were shown to have lymphadenopathy following the COVID-19 vaccine. This study assessed the patients with or without lymphadenopathy in terms of weeks, and it reported abnormal LN findings in 97% of the cases within the first day: four weeks post vaccination, persistent lymphadenopathy was reported after five to six weeks in two cases [16]. Similarly, the results of our data show an association between the duration of being vaccinated and presenting with abnormal LNs. All the patients who received the vaccine in less than four weeks presented with abnormal LNs.

In addition, the current study found that 700 cases (59.2%) of LN changes were ipsilateral to the injection site. This resonates with the findings from a similar study conducted by Cocco et al., which included 24 patients. The study found that the COVID-19 vaccine can induce axillary and supraclavicular lymphadenopathy, which was identified in the same site as the injected vaccine [15]. Moreover, one of the purposes of the present study is to assess the need for performing LN biopsy to differentiate between malignancy and vaccine-related changes.

Only 143 (12.1%) patients underwent a biopsy on account of suspicious findings on US imaging. Previous literature by Science Direct, which evaluated a total of 42 full-text articles, shows that the degree of suspicion of such lymphadenopathies is considered to be low, with only 10 biopsies performed (1.3%), and that there is little need for further workup [24]. Furthermore, the previously mentioned study that evaluated 433 participants for COVID-19 vaccine-related axillary lymphadenopathy is compatible with the present study in the gender inclusion criteria, which included both females and males. The study showed a larger number of affected females than males (29% versus 17%, respectively) [10]. Similarly, a significant percentage of 39.1% of females was affected, compared to a male percentage, which was 22.2% in the current study. Finally, in another previously mentioned study that reviewed 68 cases, the pathological findings on US included LN enlargement with diffuse or focal cortical thickening, which was reported in 29 (42.6%) cases in the literature, unlike the present study, which predicts a cortical thickening more than 0.3 cm in 321 cases (35.2%) of the cases with lymphadenopathy [16].

Limitations and recommendations

As for the limitations of this study, although the sample size was large, the study was conducted in a single center (KFUH). In addition, different types of vaccines were not considered in this study. Pfizer-BioNTech, Oxford-AstraZeneca, and Moderna vaccines were given in Saudi Arabia, and it appears from several studies that there is a difference in the prevalence of lymphadenopathy following each type of vaccine. Moreover, the majority of our sample was female cases, with only four male cases. Additionally, the majority of participants did not follow up (1,030) in order to assess the lymphadenopathy progression and change over time. Furthermore, the study did not specify the timing of the follow-up, as studies have found that lymphadenopathy following the COVID-19 vaccine resolves within 12 weeks [7]. COVID-19 vaccine is a relatively new and wide area of research; so for future research on the subject of lymphadenopathy post COVID-19 vaccine, we suggest the following: to include different types of COVID-19 vaccine, as the prevalence varies among them.

Researchers can also explore other predisposing factors for lymphadenopathy post COVID-19 vaccine. In addition, the prevalence of lymphadenopathy among those who received one, two, and three doses of the same vaccine can be researched.

## Conclusions

To sum up, this retrospective study aimed to find any possible lymphadenopathy after COVID-19 vaccination and to detect triggering or predisposing factors, which would decrease over-investigation and unnecessary medical costs. The study results showed that 39.1% of the cases were females who met the criteria of abnormal axillary LNs, and two (100%) of the cases were detected in less than four weeks following the vaccination.

Moreover, patient gender and duration after receiving the COVID-19 vaccine were the only significant associated factors. Finally, educating healthcare workers on the association of LN changes and the duration after COVID-19 vaccination will be beneficial for decreasing unnecessary investigations.
